# New transitional fossil snakeflies from China illuminate the early evolution of Raphidioptera

**DOI:** 10.1186/1471-2148-14-84

**Published:** 2014-04-18

**Authors:** Xingyue Liu, Dong Ren, Ding Yang

**Affiliations:** 1 Department of Entomology, China Agricultural University, Beijing 100193, China; 2 College of Life Sciences, Capital Normal University, Beijing 100048, China

**Keywords:** Raphidioptera, Systematics, Fossil, Middle Jurassic, China

## Abstract

**Background:**

Raphidioptera (snakeflies) is a holometabolous order of the superorder Neuropterida characterized by the narrowly elongate adult prothorax and the long female ovipositor. Mesozoic snakeflies were markedly more diverse than the modern ones are. However, the evolutionary history of Raphidioptera is largely unexplored, as a result of the poorly studied phylogeny among fossil and extant lineages within the order.

**Results:**

In this paper, we report a new snakefly family, Juroraphidiidae **fam. nov.**, based on exquisitely preserved fossils, attributed to a new species *Juroraphidia longicollum***gen.** et **sp. nov.**, from the Jiulongshan Formation (Middle Jurassic) in Inner Mongolia, China. The new family is characterized by an unexpected combination of plesiomorphic and apomorphic characters of Raphidioptera. Based on our phylogenetic analysis, Juroraphidiidae **fam. nov.** together with Raphidiomorpha form a monophyletic clade, which is the sister to Priscaenigmatomorpha. The snakefly affinity of Priscaenigmatomorpha is confirmed and another new family, Chrysoraphidiidae **fam. nov.**, is erected in this suborder.

**Conclusions:**

Juroraphidiidae **fam. nov.** is determined to be a transitional lineage between Priscaenigmatomorpha and Raphidiomorpha. Diversification of higher snakefly taxa had occurred by the Early Jurassic, suggesting that these insects had already had a long but undocumented history by this time.

## Background

Raphidioptera (snakeflies) is a distinctive, minor holometabolous order belonging to the superorder Neuropterida distinguished by the prognathous adult head, the narrowly elongate adult prothorax, and the long female ovipositor. Extant snakeflies consist of 33 genera and 240 species, all of which are placed in only two families, Raphidiidae and Inocelliidae [1,2]. Snakeflies are generally entomophagous at both larval and adult stages, although the adults of Inocelliidae have not been observed to feed, while some adult snakeflies are reported to feed on pollen [3]. Two factors that are considered to be prerequisites for the occurrence of extant snakeflies are arboreal biotopes and a climate characterized by markedly low temperatures [3,4]. Therefore, extant snakeflies are mainly distributed in the Holarctic region where typically cold winters occur, while there are a few species in the Oriental region and Central America in some high-altitude mountainous areas [3].

It is remarkable that snakeflies were much more diverse in the Mesozoic Era, with 34 genera and ca. 90 species in four extinct families: Priscaenigmatidae, Baissopteridae, Mesoraphidiidae, and Metaraphidiidae from Eurasia, North America, and South America known from fossils and specimens in amber [5-11]. A significant extinction of snakeflies at the end of the Cretaceous has been proposed to explain the reduction in diversity of families and genera as well as the contraction of their global distribution, e.g., the absence of modern snakeflies from the Southern Hemisphere [3]. Obviously, the insect paleofauna of the Paleocene is still poorly known [12], especially in the Southern Hemisphere where snakeflies might have been as abundant as in the Mesozoic Era. However, despite of scarcity, as the taphonomic control Neuroptera are recorded in several intensively explored Paleocene formations from the Southern Hemisphere [13], possibly verifying the extinction of snakeflies in this region by the end of the Cretaceous.

The phylogenetic relationships among extant and fossil taxa (especially the Mesozoic snakeflies) within Raphidioptera have been poorly studied, without any rigorous analysis using modern cladistic approaches, leaving the evolutionary history of this enigmatic insect group largely unexplored. There are two main problems to be addressed concerning the phylogeny of Raphidioptera. First, the snakefly affinity of Priscaenigmatomorpha, which is considered to be the basalmost snakefly taxon and forms a suborder of Raphidioptera [5], is unconfirmed because no species of Priscaenigmatomorpha with typical snakefly traits (i.e. the more or less narrowly elongate adult prothorax and long female ovipositor) have been found thus far. Second, the interfamilial phylogeny of Raphidioptera is unresolved, with monophyly of several extinct families untested despite attempts by Ren and Hong [14], Willmann [15], and Bechly and Wolf-Schwenninger [8].

Despite the rich diversity of snakeflies during the Cretaceous, Raphidioptera were relatively rare in the Jurassic, with only 7 genera and 14 species in the Priscaenigmatidae, Mesoraphidiidae, and Metaraphidiidae. In the Early Jurassic only Priscaenigmatidae and Metaraphidiidae are recorded, while only Mesoraphidiidae is known in the Middle and Late Jurassic. In this paper, we describe a remarkable new snakefly genus and species, *Juroraphidia longicollum***gen.** et **sp. nov.**, from the Middle Jurassic Jiulongshan Formation of Daohugou (Inner Mongolia, China), on the basis of which we erect the Juroraphidiidae **fam. nov.** as a new family of Raphidioptera, because of its markedly different morphological characters from the other known families. A phylogenetic analysis was performed to reconstruct the relationships among snakefly families and to investigate the phylogenetic status of the new family. The new snakefly family is demonstrated to be a transitional lineage between the two known suborders of Raphidioptera, which improves our understanding of the early evolution of this archaic insect order.

## Results

### Systematic palaeontology

Order Raphidioptera Navás, 1916

Family Juroraphidiidae **fam. nov.**

#### *Type genus*

*Juroraphidia***gen. nov.**

#### Diagnosis

**Figure 1:**
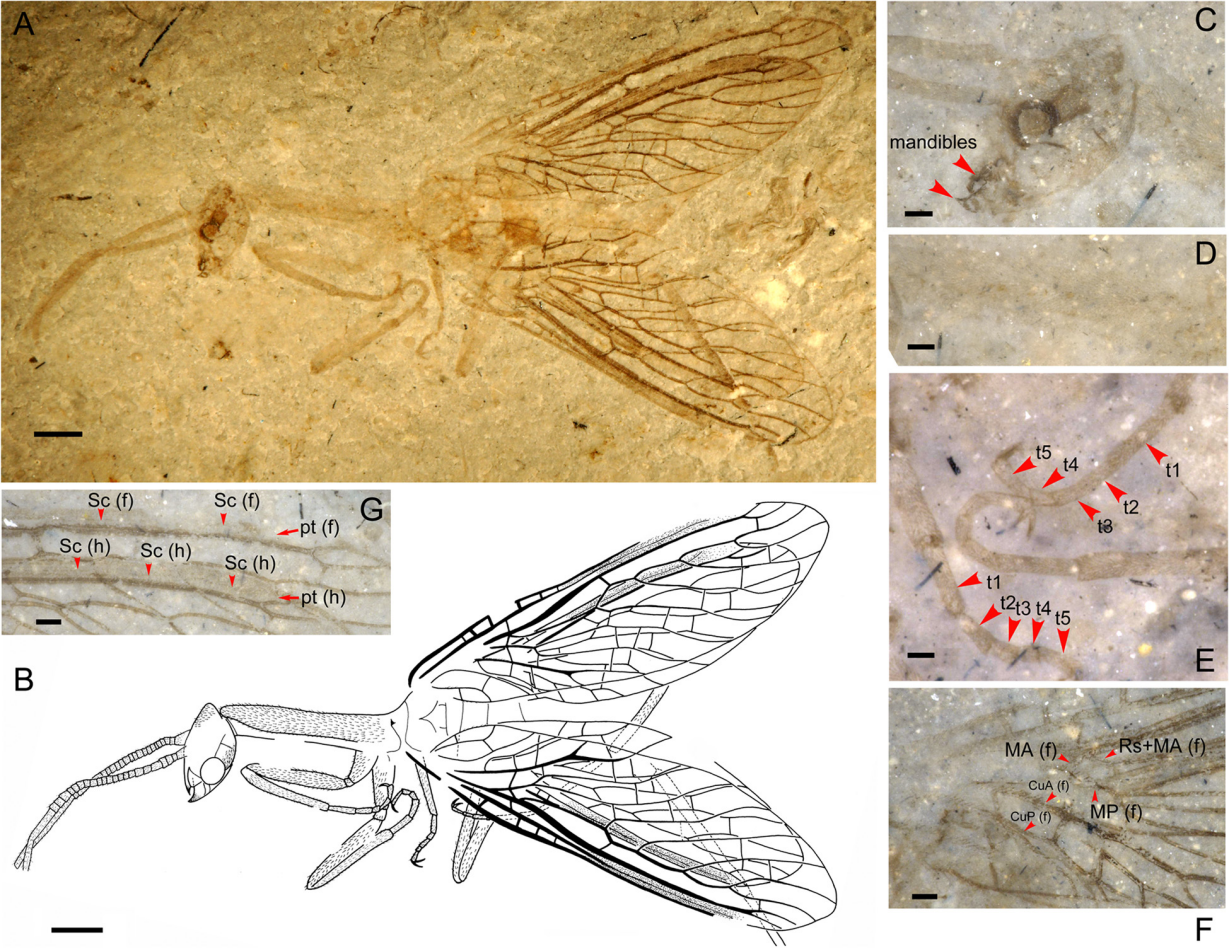
***Juroraphidia longicollum *gen. et sp. nov., holotype BMNHC-PI004804-a/b. A**, Habitus photograph; **B**. Habitus drawing; **C**, Detail of head; **D**, Detail of prothorax; **E**, Detail of tarsi; **F**, Proximal half of wings; **G**, Pterostigmatic areas of wings. t1-5, 1^st^-5^th^ tarsomere; Sc (f) and Sc (h), forewing and hindwing subcosta; pt (f) and pt (h), forewing and hindwing pterostigma; Rs + MA (f), forewing Rs + MA; MA (f), forewing media anterior; MP (f), forewing media posterior; CuA (f), forewing cubital anterior; CuP (f), forewing cubital posterior. Scale bars represent 1.0 mm in **A** and **B**; 0.2 mm in all other panels.

**Figure 2:**
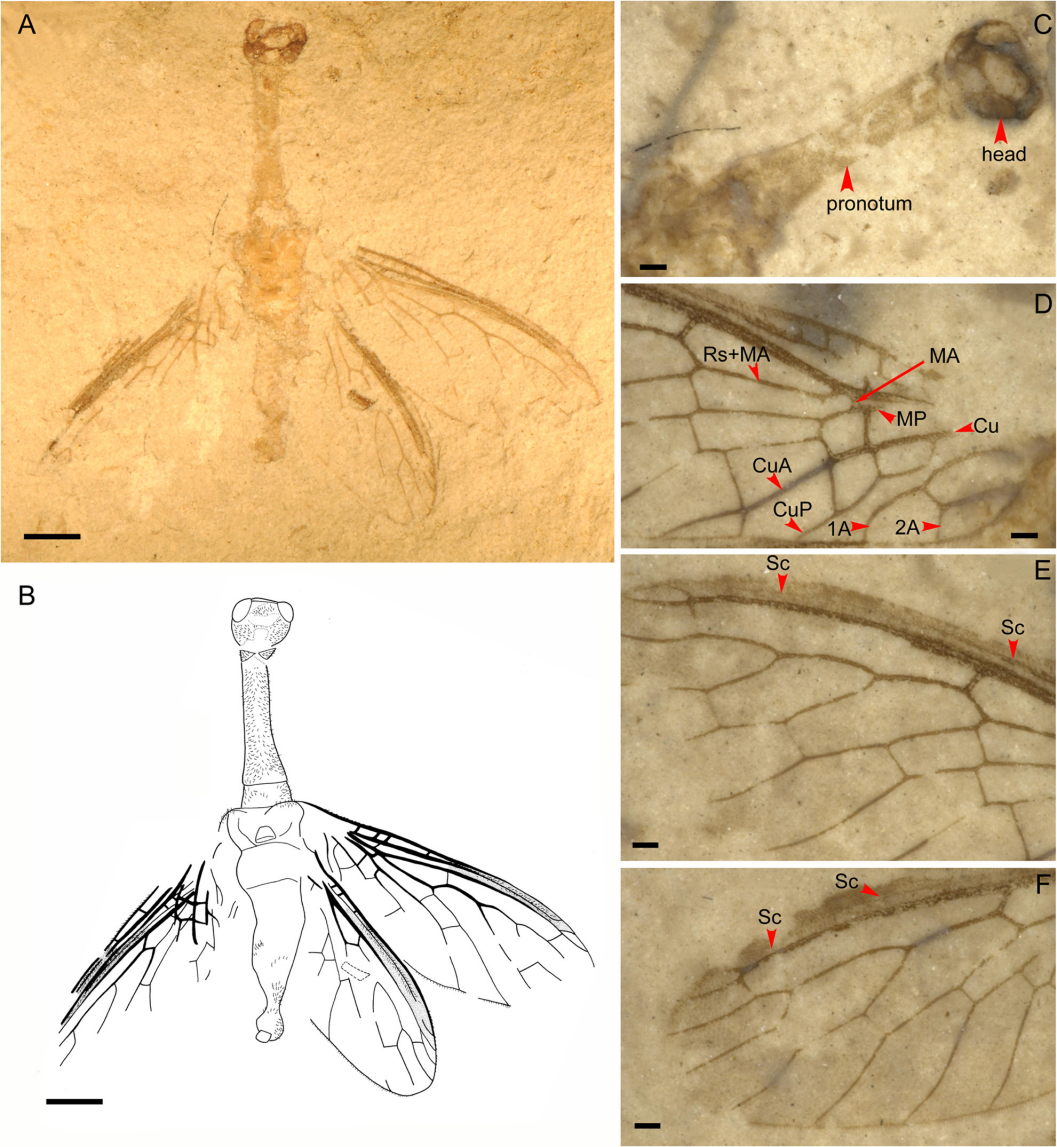
***Juroraphidia longicollum *gen. et sp. nov., paratype CNU-RAP-NN-2013001p/c. A**, Habitus photograph; **B**. Habitus drawing; **C**, Detail of head and prothorax; **D**, Proximal half of forewing; **E**, Distal half of forewing; **F**, Distal half of hindwing. Scale bars represent 1.0 mm in **A** and **B**; 0.2 mm in all other panels.

Body narrowly elongate, overall with dense and short hairs (Figure 1B and Figure 2B). Head (Figure 1B and Figure 2B) ovoid, feebly narrowed posteriorly. Prothorax (Figure 1B and Figure 2B) narrowly elongate, nearly twice as long as head, and much longer than meso- plus metathorax. Tarsi (Figure 1E) with five normal segments, 3^rd^ tarsomere not bilobed. Wings (Figure 1B and 2B) ovoid, narrowly elongate, with narrow costal regions; pterostigma distinct, elongate, open proximally, closed distally by a veinlet of R; Sc long, running within pterostigma, terminating near distal ending of pterostigma by weak fusion with C; Rs + MA origins from R near wing base; forewing MA simple, proximally presents as a short veinlet between R and stem of MP; forewing M with stem fused with R and MP deeply branched into two long, simple branches, between which only one crossvein present; CuA and CuP having a distinct common stem, forewing CuA bifurcated near wing margin; 1A short, simple, proximal half of forewing 1A arcuately curved, forming an ovoid anal cell with 2A; 2A bifurcated.

#### Autapomorphies

Among the above diagnostic characters, autapomorphies recovered in the present phylogenetic analysis for the new family are: the forewing MP with two simple main branches, the rather narrow costal region, the long pterostigma, and the presence of only one discoidal cell between main branches of MP.

#### Remarks

*Juroraphidia***gen. nov.** obviously belongs to Raphidioptera by having the typical body plan of this order, e.g. the prognathous head, the narrowly elongate prothorax anterior to fore coxae, and the distinct pterostigma. The new genus resembles Priscaenigmatomorpha based on the following character states: the long Sc extending into pterostigma, the Rs + MA separating from R near the wing base, the sparsely branched MP, and the similar configuration of CuA and CuP. Besides *Juroraphidia***gen. nov.**, there are other three genera of Raphidioptera having long Sc extending into pterostigma, namely *Hondelagia* Bode, 1953 from the Early Jurassic of England, *Priscaenigma* Whalley, 1985 from the Early Jurassic of Germany, and *Chrysoraphidia* Liu, Makarkin, Yang & Ren, 2013 from the Early Cretaceous of China, which are placed in Priscaenigmatomorpha. The diagnosis of Priscaenigmatomorpha includes the following forewing character states: Sc is long, running within the pterostigma, extending nearly to the wing apex; Rs + MA originates near the wing base; MP is basally fused with Rs + MA; Cu is continuous with CuA; the cell between 1A and 2A is narrow [11]. *Hondelagia* and *Priscaenigma* have the above typical characters of Priscaenigmatomorpha and comprise the family Priscaenigmatidae. However, *Chrysoraphidia* is tentatively placed into Priscaenigmatomorpha but not assigned to Priscaenigmatidae because this genus has the forewing MP fusing with neither Rs + MA nor CuA, the Rs branches being not zigzagged, and the pectinate forewing 1A [11].

The synapomorphies of Priscaenigmatomorpha were proposed to be the fusion between Sc and R at least on forewings, the narrowly elongate cell between forewing R and Rs, the similar cell pattern in distal half of wings, and the unbranched or only apically forked forewing MP, by Bechly and Wolf-Schwenninger [8] based on the examination of *Hondelagia* and *Priscaenigma*. However, if we accept the placement of *Chrysoraphidia* into Priscaenigmatomorpha, the characters (the fusion between Sc and R at least on forewings, the narrowly elongate cell between forewing R and Rs, and the similar cell pattern in distal half of wings) can only be interpreted as the synapomorphies of Priscaenigmatidae, and the branching pattern of forewing MP is the single character shared by the three genera of Priscaenigmatomorpha although it is also probably plesiomorphic.

After careful consideration, *Juroraphidia***gen. nov.** can be assigned to neither Priscaenigmatidae nor a same family with *Chrysoraphidia* by having the extremely elongate pterostigma, the forewing Sc not fused with R (shared by *Chrysoraphidia*), the reduction of crossveins between R and Rs, the presence of only one gradate crossveins series, the branching pattern of CuA, and the ovoid forewing anal cell between 1A and 2A. Based on the long Sc, the distinctly proximal separating point of Rs + MA from R, the forewing M proximally fused with R, and the adult 3^rd^ tarsomeres not bilobed, *Juroraphidia***gen. nov.** also greatly differs from all families of Raphidiomorpha although similar ovoid forewing anal cell between 1A and 2A is shared by *Juroraphidia***gen. nov.** and Raphidiomorpha. Therefore, it is firm to erect a new family of Raphidioptera based on *Juroraphidia***gen. nov**.

### Genus *Juroraphidia* gen. nov

#### Type species

*Juroraphidia longicollum***sp. nov.**

#### Derivation of name

The generic name is after the geological period ‘Jurassic’, and *Raphidia*, a common suffix for Raphidioptera. Gender: feminine.

#### Diagnosis

Small-sized raphidiopterans (forewing 6.1-8.6 mm long). Other as family characters.

#### Remarks

Previously, only genera of Mesoraphidiidae of Raphidiomorpha are known from the Middle Jurassic and all of them are recorded from China. The new genus represents the first record of a different family other than Mesoraphidiidae and enriches our knowledge on the diversity of Raphidioptera from the Middle Jurassic.

### *Juroraphidia longicollum* sp. nov.

#### Derivation of name

The specific epithet is derived from Latin *longi-*, long, and *Collum*, neck, in reference to the distinctly long prothorax in this species.

#### Type materials

Holotype, BMNHC-PI004804-a/b (part and counterpart), a well-preserved specimen in dorsal aspect (BMNH). Paratype, CNU-RAP-NN-2013001p/c (part and counterpart), a well-preserved specimen in dorsal aspect (CNU).

#### Type locality and horizon

All collected from the Daohugou locality (41°18’30”N, 119°13’00”E), Ningcheng County, Neimenggu Autonomous Region [Inner Mongolia], China; Middle Jurassic (Jiulongshan Formation).

#### Diagnosis

As for the genus.

#### Description

**Figure 3:**
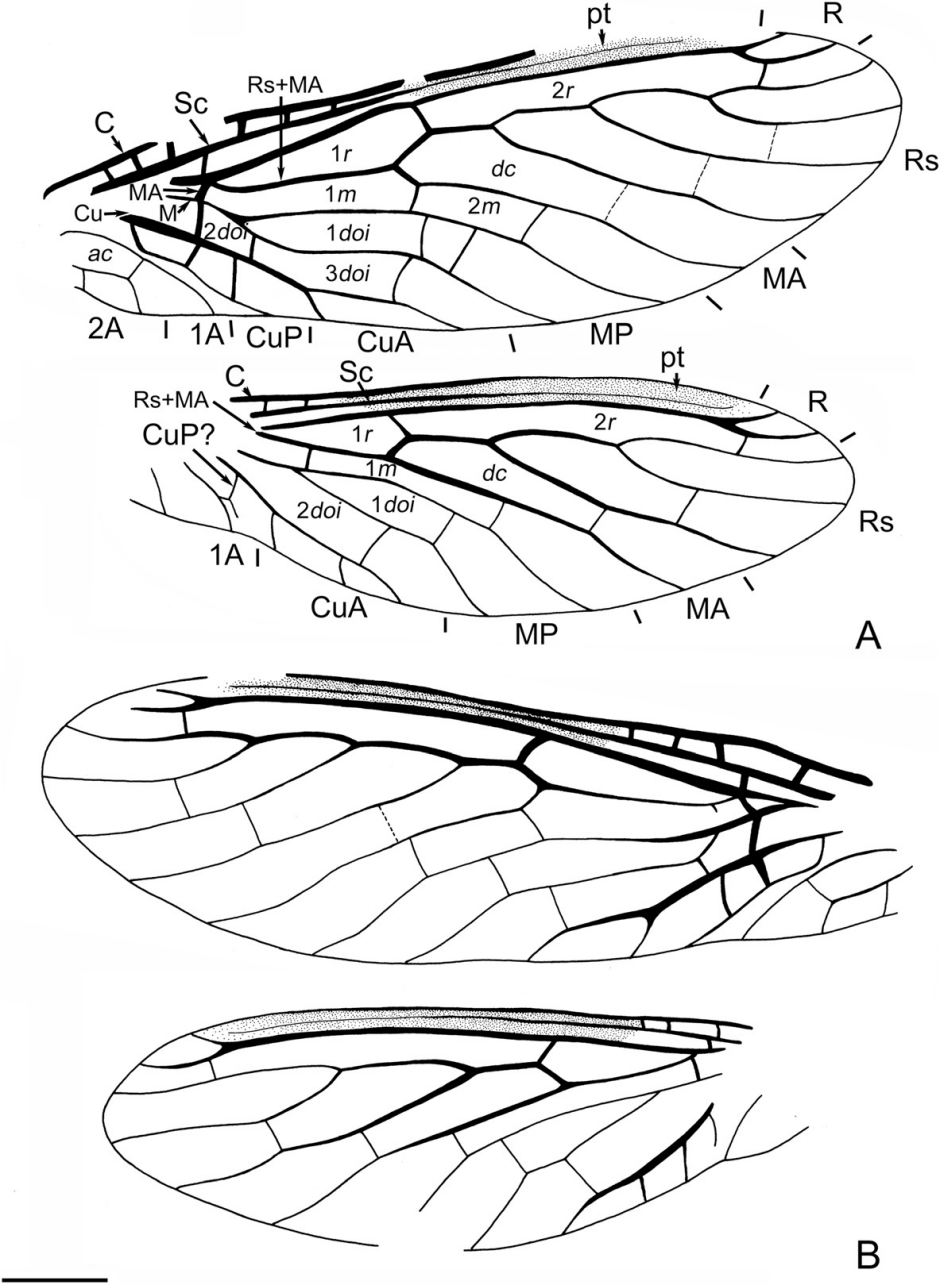
***Juroraphidia longicollum *gen. et sp. nov., holotype BMNHC-PI004804-a. A**, Fore- and hindwing; **B**, Fore- and hindwing, opposite set. Scale bar represents 1.0 mm.

*Holotype* (Figure 1 and Figure 3). Body well preserved except for metathorax and abdomen. Head (Figure 1C) length 2.1 mm, ovoid, feebly narrowed posteriorly, densely covered by short setae at least on postocular portions; compound eye preserved in lateral view, ovoid; ocelli not observed; mouthparts with labrum and mandibles preserved, labrum triangular, mandibles subtriangular with apices slightly incurved and acutely produced; antennae filiform, with at least 30 segments, nearly as long as pronotum, densely covered by short setae, scape subcylindrical and much larger than remaining segments. Prothorax (Figure 1D) preserved in lateral view, narrowly elongate, nearly twice as long as head; pronotum 3.1 mm long, densely covered by short seate, with lateral portions not curving ventrally. Mesothorax robust; 1.3 mm long, 1.8 mm wide. Legs slenderly elongate, densely covered by short setae; hind leg much longer than fore and hind legs; tarsi (Figure 1D) with five tarsomeres and two tarsal claws, 1^st^ tarsomere much longer than each of 2^nd^ to 5^th^ tarsomeres, 3^rd^ tarsomere not bilobed.

Forewing (Figure 3) ovoid, narrowly elongate; 8.9 mm long, 2.7 mm wide. Costal area nearly as wide as subcostal area, and much more narrowed on pterostigmatic area, proximally with 5–6 veinlets preserved. Sc long, running within pterostigma with same distance to C and Sc, terminating near distal ending of pterostigma by weak fusion with C. R long, terminating before wing apex, distally with one short and one longer simple veinlets. One sc-r present at proximal 1/4. Pterostigma well developed, elongate (ca. 4.2 mm long), nearly half of forewing length, dark. Two crossveins present between R and Rs, forming two elongate radial cells; 2*r* about 1.5 times as long as 1*r*. Rs + MA origins from R approximately 1.6 mm from wing base. Rs with four simple branches. One gradate crossveins series present between branches of Rs as well as between posterior branch of Rs and MA, one large and elongate discal cell (*dc*) present. MA proximally present as a short veinlet between R (at branching point of Rs + MA from R) and stem of MP. Two crossveins present between MA and MP, forming two medial cells (1-2*m*); 1*m* about twice length of 2*m*. Stem of M fused with R; MP deeply branched into two long simple branches. One or two discoidal cells (*doi*) present between branches of MP; two *doi* present between MP and CuA. Cu deeply dividing into CuA and CuP, rather proximal to origin of Rs + MA and M; CuA bifurcated near wing margin; CuP simple, with base arcuately curved. Two crossveins present between CuA and CuP. 1A short, simple, proximal half arcuately curved, forming an ovoid anal cell with 2A. 2A bifurcated, with anterior branch angulately curved distad. Membrane probably colourless, transparent except for dark pterostigma.

Hindwing (Figure 3) much shorter than forewing, with anal area distinctly narrowed; 7.2 mm long, 2.3 mm wide. Costal area narrow, and much narrower on pterostigmatic area, proximally with 2–3 veinlets preserved. Sc long, running within pterostigma with same distance to C and Sc, terminating near distal ending of pterostigma by weak fusion with C. R long, terminating before wing apex, distally with one short and one longer simple veinlets. One sc-r present quite near wing base. Pterostigma well developed, elongate (ca. 2.5 mm long), more than half of forewing length, dark. Two crossveins present between R and Rs, forming two elongate radial cells; 2*r* about 3.0 times as long as 1*r*. Rs + MA origins from R approximately 3.6 mm from wing base. Rs with three simple branches. One crossvein present between anterior and posterior branches of Rs; one crossveins present between proximal branch of Rs and MA, forming one large and elongate discal cell (*dc*). Basal part of MA not preserved. One distal crossvein present between MA and MP, forming a rather narrow and elongate medial cell (1*m*). Basal part of MP not preserved; MP proximally branched into two long simple branches. One small discoidal cell (1*doi*) present between branches of MP; one (2*doi*) present between MP and CuA, about 1.5 times as long as 1*doi*. CuA trifurcated near wing margin; CuP possibly separated from Cu much more distad but not reaching wing margin. 1A short, simple. 2A not preserved. Membrane probably colourless, transparent except for dark pterostigma.

**Figure 4:**
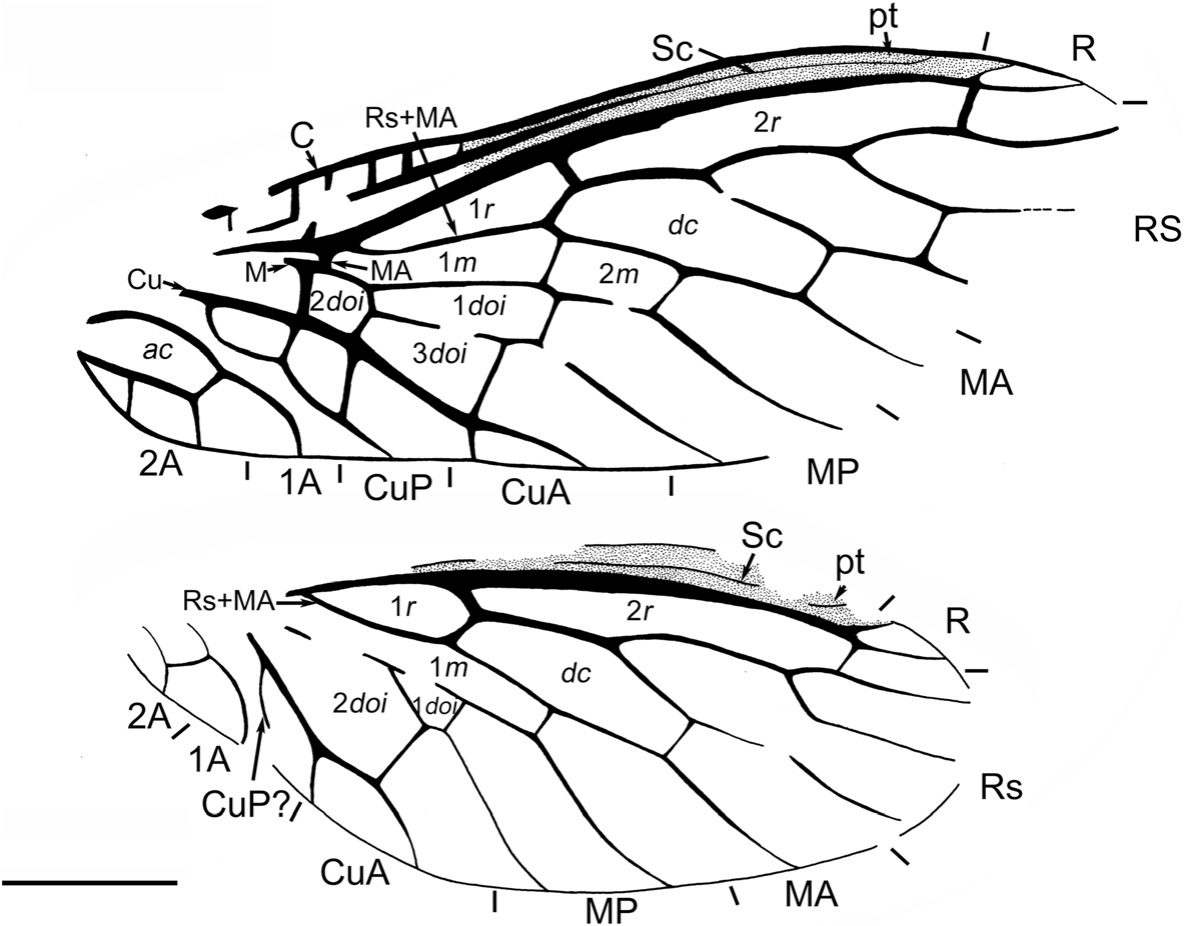
***Juroraphidia longicollum *gen. et sp. nov., paratype CNU-RAP-NN-2013001p.** Fore- and hindwing. Scale bar represents 1.0 mm.

*Paratype* (Figure 2 and Figure 4). Body (Figure 2A) narrowly elongate, with dense and short hairs; body length 8.0 mm. Head (Figure 2C) ovoid, narrowed posteriorly, with a pair of elliptical compound eyes, mouthparts and antennae not preserved, ocelli not observed; head width 1.1 mm. Prothorax (Figure 2C) narrowly elongate, much longer than meso- plus metathorax, slightly broadened posteriorly at posterior 1/3; 2.7 mm long, 0.5 mm wide. Meso- and metathorax robust; 1.6 mm long, 1.2 mm wide. Legs not preserved. Abdomen (Figure 2A,B) narrower than metathorax (2.5 mm long, 0.9 mm wide at maximum in dorsal view), gradually narrowed posteriad; abdominal segments invisible; abdominal apex slightly inflated, terminally with a subquadrate sclerite, which is probably ectoproct.

Forewing (Figure 4) ovoid, narrowly elongate; 6.1 mm long, 2.2 mm wide. Costal area narrow, and much narrower on pterostigmatic area, proximally with six veinlets preserved. Sc long, running within pterostigma with same distance to C and Sc, terminating near distal ending of pterostigma by weak fusion with C. R long, terminating before wing apex, distally with one short and one longer simple veinlets. One sc-r present at proximal 1/4. Pterostigma well developed, elongate (2.7 mm long), nearly half of forewing length, dark. Two crossveins present between R and Rs, forming two elongate radial cells (1-2*r*); 2*r* about twice length of 1*r*. Rs + MA origins from R approximately 1.0 mm from wing base. Rs with three simple branches. One crossvein present between anterior and posterior branches of Rs; one crossveins present between posterior branch of Rs and MA, forming one large and elongate discal cell (*dc*). MA proximally present as a short veinlet between R (at branching point of Rs + MA from R) and stem of MP. Two crossveins present between MA and MP, forming two medial cells (1-2*m*); 1*m* about twice of 2*m* in length. Proximal part of M not completely preserved, but with stem rather approaching to R; MP deeply branched into two long simple branches. One discoidal cell (1*doi*) present between branches of MP; two (2*doi* and 3*doi*) present between MP and CuA. Cu deeply dividing into CuA and CuP, rather proximal to origin of Rs + MA and probably also proximal to origin of M; CuA bifurcated near wing margin; CuP simple. Two crossveins present between CuA and CuP. 1A short, simple, proximal half arcuately curved, forming an ovoid anal cell with 2A. 2A bifurcated, with anterior branch angulately curved distad. Membrane probably colourless, transparent except for dark pterostigma.

Hindwing (Figure 4) much shorter than forewing, with anal area distinctly narrowed; 4.9 mm long, 1.7 mm wide. Costal area narrow, and much narrower on pterostigmatic area, proximally with three veinlets preserved. Sc long, running within pterostigma with same distance to C and Sc, terminating near distal ending of pterostigma by weak fusion with C. R long, terminating before wing apex, distally with one short and one longer simple veinlets. sc-r not preserved. Pterostigma well developed, elongate (ca. 2.5 mm long), more than half of forewing length, dark. Two crossveins present between R and Rs, forming two elongate radial cells (1-2*r*); 2r about 3.0 times as long as 1r. Rs + MA originates from R approximately 0.7 mm from wing base. Rs with three simple branches. One crossvein present between anterior and posterior branches of Rs; one crossveins present between posterior branch of Rs and MA, forming one large and elongate discal cell (*dc*). Basal part of MA not preserved. One distal crossvein present between MA and MP, forming a rather narrow and elongate medial cell (1*m*). Proximal part of MP not preserved; MP branched at its mid length into two long simple branches. One small discoidal cell (1*doi*) present between branches of MP; one (2*doi*) present between MP and CuA, about 6.0 times as large as 1*doi*. CuA bifurcated near wing margin; CuP possibly separated from Cu much more distad but not reaching wing margin. 1A short, simple, arcuately curved posteriorly near wing margin. 2A simple. Membrane probably colourless, transparent except for dark pterostigma.

#### Remarks

The holotype of *J. longicollum***sp. nov.** differs from the paratype of same species by the slightly larger body-size and the forewing Rs with four branches, while the paratype of *J. longicollum***sp. nov.** is much smaller and has the forewing Rs bearing three branches. However, all diagnostic characters of *J. longicollum***sp. nov.** can be found in these two specimens. Moreover, the intraspecific variation of the branching pattern of Rs is common in Raphidioptera. Therefore, we consider these two specimens to be conspecific. The paratype of *J. longicollum***sp. nov.** is probably a male because its abdominal apex is slightly inflated, resembling the male genital segments in extant snakeflies, and lacks the elongate ovipositor. Due to lacking of abdomen, we cannot presume the sex of the holotype of *J. longicollum***sp. nov.**

### Phylogenetic analysis

**Figure 5:**
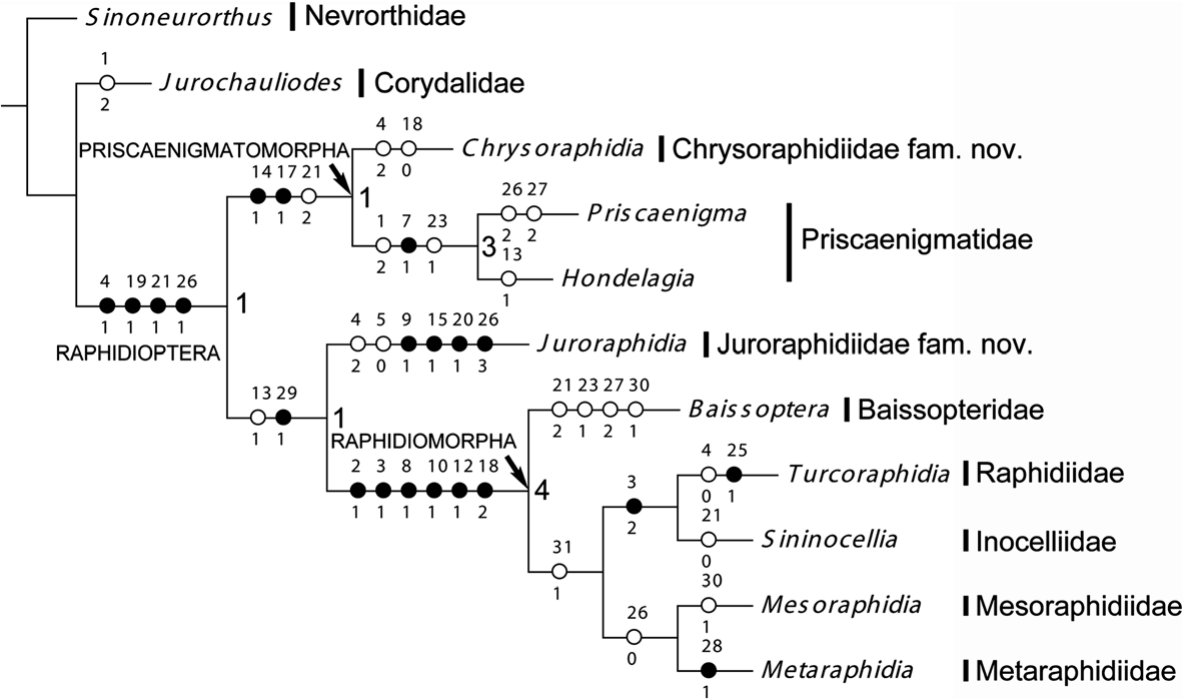
**Phylogenetic relationships among families of Raphidioptera.** Single most parsimonious tree obtained by NONA. Bremer support values mapped at nodes, unambiguous apomorphies mapped on branches, black circles indicate nonhomoplasious changes.

Phylogenetic analysis using NONA yielded only one most parsimonious tree (MPT) (length = 64, consistency index = 64, retention index = 72) (Figure 5). Raphidioptera is confirmed to be monophyletic. Within Raphidioptera, the monophyly of Priscaenigmatomorpha and Raphidiomorpha is also confirmed. Juroraphidiidae **fam. nov.** is assigned to be the sister-group of Raphidiomorpha. Priscaenigmatomorpha is recovered as the sister-group of the clade consisting of Juroraphidiidae **fam. nov.** and Raphidiomorpha. Within Priscaenigmatomorpha, *Hondelagia* and *Priscaenigma* form a monophyletic group, supporting the monophyly of Priscaenigmatidae. Within Raphidiomorpha, Baissopteridae is recovered as the sister-group of the remaining four families, which are split into two lineages, Mesoraphidiidae + Metaraphidiidae and Raphidiidae + Inocelliidae.

Phylogenetic analysis using TNT yielded 4 MPTs, in which the interfamilial relationships within Raphidiomorpha are different. One of the 4 MPTs has an identical topology to the single MPT obtained from the analysis with NONA. The strict consensus tree of the 4 MPTs is shown in Additional file 1: Figure S1. Despite the poorly resolved interfamilial phylogeny within Raphidiomorpha, the relationships among Priscaenigmatomorpha, Juroraphidiidae **fam. nov.**, and Raphidiomorpha are consistent with the results of the NONA analysis.

## Discussion

### Snakefly affinity of Priscaenigmatomorpha

Snakeflies are easily recognizable because of their unique adult morphological traits: an ovoid prognathous head, an elongate prothorax, and a long, blade-like female ovipositor. Excluding Priscaenigmatomorpha from Raphidioptera, the autapomorphies of Raphidioptera proposed by Aspöck and Aspöck [16] are: (1) the forewing Sc running into the anterior wing margin (i.e., Sc is very short), (2) imaginal tarsi with expanded (bilobed) third tarsomeres, (3) amalgamation of male tergite 9 and sternite 9 as a ring, and (4) elongation of the female ovipositor. On the other hand, the snakefly affinity of Priscaenigmatomorpha was agreed by Willmann [15], Engel [5], Bechly and Wolf-Schwenninger [8], and Liu et al. [11], although all autapomorphies proposed by Aspöck and Aspöck [16] for Raphidioptera have not been found in Priscaenigmatomorpha, in which the forewing Sc is obviously long and the other three characters are not preserved in the currently known fossils. Thus, the placement of Priscaenigmatomorpha in Raphidioptera is based on only one possible synapomorphy, i.e., the pterostigma being at least weakly developed [11]. However, convincing evidence supporting the snakefly affinity of Priscaenigmatomorpha has been scarce before our present findings.

The family Juroraphidiidae **fam. nov.** described herein appears to be a transitional lineage between Priscaenigmatomorpha and Raphidiomorpha. The overall body plan, especially the prognathous head and rather elongate prothorax, in Juroraphidiidae **fam. nov.** indicates that the new family definitely belongs to Raphidioptera. The ovoid forewing anal cell (char. 29:1) shared by Juroraphidiidae **fam. nov.** and Raphidiomorpha is recognized as a synapomorphy of these two groups in our phylogenetic analysis. Furthermore, the similar wing venation of Juroraphidiidae **fam. nov.** and Priscaenigmatomorpha—the long forewing Sc, the rather proximally originating Rs + MA, the forewing M proximally fused with R but not CuA, and the forewing CuA and CuP having a common stem—provides evidence for the snakefly affinity of Priscaenigmatomorpha. The configuration of these venational features combined with a distinct pterostigma is unique in Neuropterida and is only shared by Juroraphidiidae **fam. nov.** and Priscaenigmatomorpha. Although these wing venational features are plesiomorphic, the retention of these characters in a true snakefly greatly improves our understanding of the morphological evolution of Raphidioptera. Therefore, there is no reason to exclude Priscaenigmatomorpha from Raphidioptera only because of the lack of a short forewing Sc.

Based on the results of our phylogenetic analysis, the synapomorphic characters of Raphidioptera are the medially forked forewing MA (char. 4:1), the distinct forewing pterostigma (char. 19:1), the presence of only two forewing radial cells (char. 21:1), and the presence of three forewing discoidal cells between the main branches of MP (char. 26:1). However, variations in the forking condition of forewing MA and the number of forewing radial and discoidal cells are observed in certain snakefly families, and the distinct pterostigma are still the only undisputed synapomorphy of Raphidioptera. Nevertheless, an elongate prothorax has been observed in Juroraphidiidae **fam. nov.** and most families of Raphidiomorpha (this feature is not known for Metaraphidiidae because no fossils possessing a preserved prothorax have been found). Thus, in Priscaenigmatomorpha the pronotum was most probably slightly elongate in *Chrysoraphidia relicta*, on the basis of the position of the head and mesothorax in a paratype of this species [11]. Therefore, if the prothorax is also elongate in Priscaenigmatidae, this feature might be another good synapomorphy for Raphidioptera, although it relies on future discovery of a well-preserved fossil of Priscaenigmatidae.

### Internal phylogeny of Raphidioptera

The monophyly of Priscaenigmatomorpha is supported by the simple forewing 2A (char. 14:1), the absence of forewing 1sc-r (char. 17:1), and the presence of four or more forewing radial cells (char. 21:2). Within this suborder, the monophyly of Priscaenigmatidae is supported by the distal fusion between forewing Sc and R (char. 1:2), the strongly zigzagged forewing Rs (char. 7:1), and the presence of many crossveins between forewing Rs branches (char. 23:1). The genus *Chrysoraphidia*, which greatly differs from Priscaenigmatidae based on the following diagnosis in possessing the long Sc with free ending, the configuration of forewing MP, the Rs not strongly zigzagged, and the pectinate forewing 1A, should represent another family in Priscaenigmatomorpha. Therefore, we herein erect another new family of Raphidioptera, Chrysoraphidiidae **fam. nov.** (type genus: *Chrysoraphidia* Liu, Makarkin, Yang & Ren, 2013; see diagnosis in Liu et al. [11]). The synapomorphy of Chrysoraphidiidae **fam. nov.** is defined to be the simple forewing MA (char. 4:2) and the position of forewing 2sc-r within the pterostigma (char. 18:0). Although the simple forewing MA is shared by Juroraphidiidae **fam. nov.**, it is better interpreted as convergent derivation, and further autapomorphies might be found after discovery of additional genera and species of Chrysoraphidiidae **fam. nov.**

A number of autapomorphies of Juroraphidiidae **fam. nov.** are recognized: the forewing MP with two simple main branches (char. 9:1), the rather narrow costal region (char. 15:1), the long pterostigma that is nearly a half of wing length (char. 20:1), and the presence of only one discoidal cell between main branches of MP (char. 26:3). As discussed above, Juroraphidiidae **fam. nov.** is assigned to be the sister of Raphidiomorpha based on the presence of an ovoid forewing anal cell, representing a lineage bridging Priscaenigmatomorpha and Raphidiomorpha. Nevertheless, it is premature to erect a new suborder of Raphidioptera based only on Juroraphidiidae **fam. nov.**, in which there is only one known genus and species.

The monophyly of Raphidiomorpha is well supported by the much more distal position of the branching points of the last forewing Rs branch, Rs + MA, and the main branches of the forewing MP (chars. 2:1, 3:1, 10:1), the forewing MP being proximally fused with CuA (char. 8:1), the parallel forewing CuA and CuP (char. 12:1), and the presence of a forewing 2sc-r proximal to the pterostigma (char. 18:2). The Raphidiomorpha undoubtedly represents the crown group of Raphidioptera with a high species diversity. However, a large number of described fossils are known as only fragmentarily preserved wings and some have been poorly described, which apparently limits the reconstruction of the interfamilial phylogeny within Raphidiomorpha. A comprehensive revision of all fossil snakefly genera and species would be desirable to clarify the classification of Raphidiomorpha, but is outwith the scope of the present paper.

The results of this study on the interfamilial relationships within Raphidiomorpha, although weakly supported in the phylogenetic analysis, show some similarity with the previous hypothesis proposed by Willmann [15] and Bechly and Wolf-Schwenninger [8]. First, Baissopteridae, irrespective of its monophyly or paraphyly, is the probable sister group of the remaining families of Raphidiomorpha. The general increase of Rs branches and radial cells, which has been considered to be plesiomorphic [8], might alternatively be the synapomorphy of this family, and evolved convergently in some groups of Priscaenigmatomorpha. Second, the sister-group relationship between Raphidiidae and Inocelliidae, which make up the infraorder Neoraphidioptera, is also recovered in our analysis with the synapomorphy of the forewing Rs + MA separated from R at the middle (char. 3:2). However, Metaraphidiidae, which was placed into Mesoraphidiidae by Engel [5] and then erected to be a new family by Bechly and Wolf-Schwenninger [8], is assigned as the sister group of Mesoraphidiidae, and Mesoraphidiidae + Metaraphidiidae is assigned as the sister lineage of Neoraphidioptera in our analysis. This differs from the hypothesis proposed by Bechly and Wolf-Schwenninger [8] that Neoraphidioptera and Metaraphidiidae are sister groups based on the proximal fusion between hindwing MA and MP, which is apparently plesiomorphic in Neuropterida. It should be noted that, despite the absence of a forewing cua-cup and the fusion of hindwing MA and MP, the venation of Metaraphidiidae is generally similar to that of Mesoraphidiidae, especially the triangular arrangement of three forewing discoidal cells. Therefore, the validity of Metaraphidiidae, together with the monophyly of Mesoraphidiidae, should be reconsidered when more fossils of these groups are discovered.

### Origin and early evolution of Raphidioptera

Raphidioptera is traditionally considered to be the sister-group of Megaloptera and had been placed as a family into the latter order [17]. The finding of the family Nanosialidae from the late Permian of Russia, originally described as an ancestral group of Raphidioptera but now placed in the order Panmegaloptera (= Megaloptera s.l.), seemingly supports a monophylum comprising Megaloptera and Raphidioptera because of the venational similarity between Nanosialidae and the snakefly family Mesoraphidiidae [18]. The proposed synapomorphies of Nanosialidae + Raphidioptera are: enlarged pterostigma, nygmata absent, wing membrane bare with short, stiff, erect setae along veins, MP fused with CuA and having more branches than Rs, short free 3A sometimes restored, and small hindwing anal area [18]. However, in Priscaenigmatomorpha and Juroraphidiidae **fam. nov.**, the fusion between the forewing MP and CuA is absent, which is probably plesiomorphic. Thus, the fusion between the forewing MP and CuA might be independently derived in the Permian Nanosialidae and the younger Raphidioptera from the Mesozoic. Additionally, the other synapomorphies above listed (the shape of the pterostigma and hindwing anal area, the absence of nygmata, and the wing membrane characteristics) are more questionable because of their presence in various heterogeneous lineages in Neuropterida. Therefore, there is still no good evidence to support Raphidioptera being the descendant of early Megaloptera.

**Figure 6:**
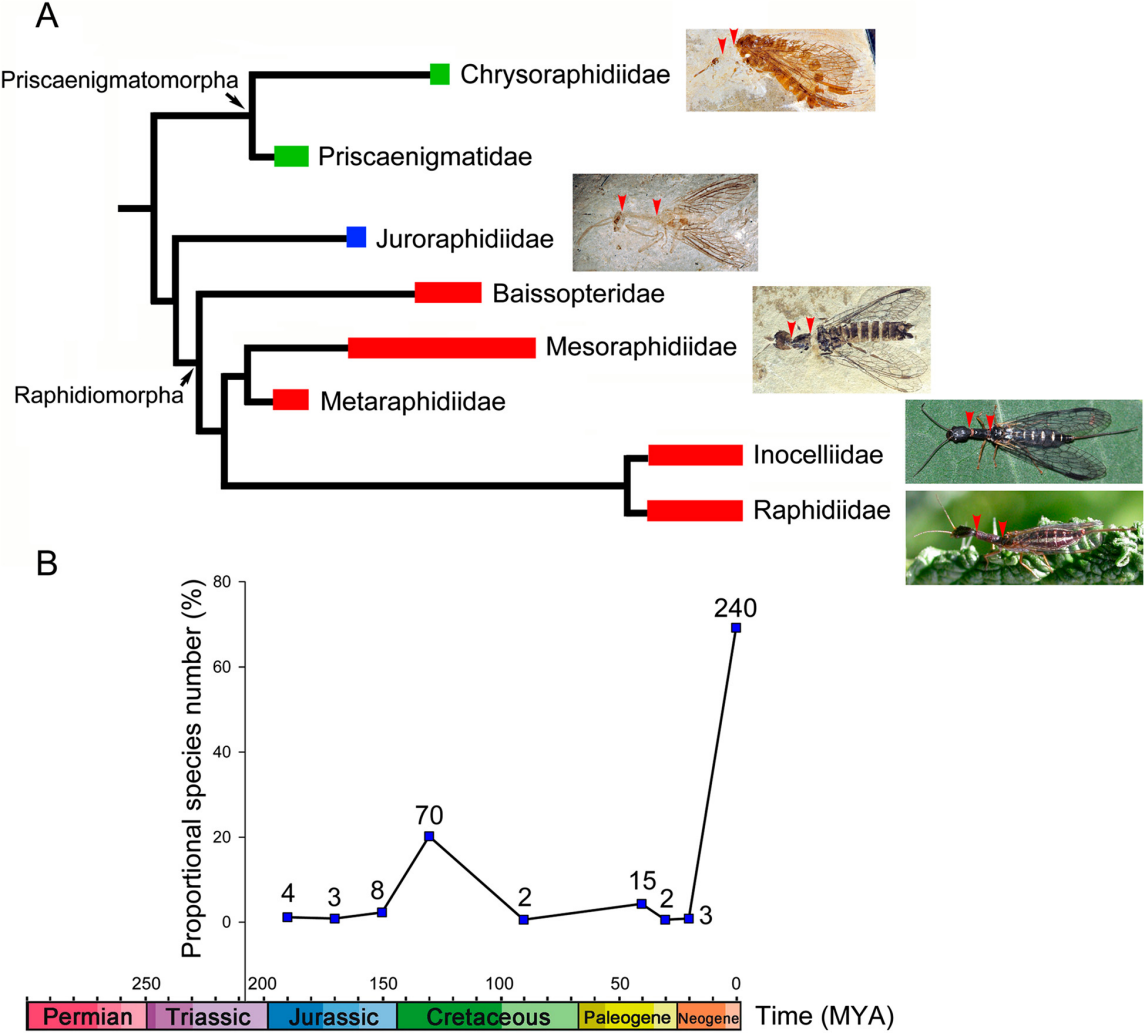
**Evolutionary chronogram of Raphidioptera. A**, Interfamilial phylogeny of Raphidioptera based on present result; broad lines indicate known geological distributions; black lines are hypothesized ranges; habitus photos of *Chrysoraphidia* (Priscaenigmatomorpha: Chrysoraphidiidae **fam. nov.**, *Juroraphidia***gen. nov.** (Juroraphidiidae **fam. nov.**), *Mesoraphidia* (Raphidiomorpha: Mesoraphidiidae), *Inocellia* (Raphidiomorpha: Inocelliidae) and *Xanthostigma* (Raphidiomorpha: Raphidiidae) are shown from top to bottom, with red arrows marked to indicate the relative length of prothorax; **B**, Plot of change of snakefly species numbers over time; proportional species number = species number in relevant geological period/total number of known extant and extinct snakefly species; species number of each geological period (i.e. Early Jurassic, Middle Jurassic, Late Jurassic, Early Cretaceous, Late Cretaceous, Eocene, Oligocene, Miocene and Holocene) shown at relevant scale.

Furthermore, an increasing number of studies using both morphological and molecular evidence have shown that Raphidioptera is the sister-group of the remaining two orders of Neuropterida, i.e. Megaloptera + Neuroptera [19-22]. The fossil record indicates that both Megaloptera and Neuroptera had originated no later than the late Permian [12]. As the putative sister-group of Megaloptera + Neuroptera, Raphidioptera should also have diverged from the stem group of Neuropterida during the late Permian, which is in good agreement with the estimated divergence time (ca. 250 Ma, late Permian) of snakeflies based on molecular clock approaches [22,23]. However, the earliest currently known fossil snakefly is of Early Jurassic age [15,24,25]. The earliest snakefly fossils are attributed to both Priscaenigmatomorpha and Raphidiomorpha. As the sister lineage of Raphidiomorpha, Juroraphidiidae **fam. nov.** should also have originated during the Early Jurassic, although it is only known from the Middle Jurassic so far. Thus, the Early Jurassic appears to be a crucial period for the diversification of snakefly suborders, which, on the other hand, indicates a probable much earlier origin of stem Raphidioptera than the Early Jurassic (Figure 6).

Considering both the historical and modern species diversity of Raphidioptera (Figure 6), the Northern Hemisphere has undoubtedly been the centre of snakefly diversification since the Early Jurassic, with ~98% of world snakefly species confined to this part of the globe. Nevertheless, it is obvious that Raphidioptera was widely distributed in Laurasia and Gondwana at least during the Early Cretaceous, and this distribution pattern was probably caused by north–south dispersal of some snakefly lineages before the breakup of Pangaea in the Late Jurassic (~155 Ma) because the Cretaceous snakefly fossils from the Southern Hemisphere show close affinity with the diverse Mesozoic snakeflies of the Northern Hemisphere, e.g., Baissopteridae is found in the Early Cretaceous of Eurasia and South America [5]. Compared with the rich diversity of Mesozoic snakeflies in the Northern Hemisphere, the Southern Hemisphere contained many fewer snakefly species. Only four species from the Early Cretaceous of Brazil are known, and its snakefly fauna became extinct, presumably as a result of the extraterrestrial impact at the end of the Cretaceous [3].

The regions that are currently known to have contained Mesozoic snakeflies had warm temperate climates. It is worth mentioning that there were two warm temperate regions located at the northern and southern mid-latitude parts of Pangaea, caused by the isolation of subtropical desert, from the early Permian to the end of the Late Jurassic [12,26]. Remarkably, all known Early Jurassic Raphidioptera, the earliest records of this order, occur only in Eurasia, which was in the northern part of Pangaea and largely a warm temperate region, and was probably favored by Mesozoic snakeflies during the Early Jurassic. Therefore, if snakeflies had been present in the southern part of Pangaea during the Early Jurassic, they were probably isolated from those in the northern part of Pangaea because of the presence of subtropical desert regions, which could prevent the north–south dispersal of snakeflies. During the Middle and Late Jurassic, the subtropical desert regions reduced in size [12,26] and snakeflies could have dispersed north–south in this period. Because the Jurassic insect fossils from the Southern Hemisphere are extremely sparse and require significant study [12], it is hard to estimate whether the austral Mesozoic snakeflies came from the northern part of Pangaea through the dispersal of their relatives from north to south. However, we cannot eliminate the possibility that snakeflies originated in the northern part of Pangaea and had not dispersed across the equator to the opposite side of globe before the Middle–Late Jurassic.

Considering the morphological evolution of Raphidioptera, before our present discoveries only a few Middle Jurassic fossils of Mesoraphidiidae with well-preserved bodies were known, and in all these species the prothorax is narrower but slightly shorter than the head [6]. This is likely to be plesiomorphic, because all known snakefly larvae as well as many adults have this feature [27]. As predatory insects, the narrow prothorax in Raphidioptera allows flexible movement of the much broader prognathous head with well-developed mandibles during hunting. A similar configuration of head and prothorax is also present in dobsonflies (Megaloptera: Corydalidae), but is frequently used for male-male combat or defense [28]. Our present finding of Juroraphidiidae **fam. nov.**, which has a narrowly elongate prothorax, demonstrates that this typical snakefly trait was already present in some basal Mesozoic snakeflies as well as Raphidiomorpha in the Middle Jurassic, and the stem group of Raphidiomorpha might have biological habits similar to those of their descendants. Moreover, it should be mentioned that the prothorax of Juroraphidiidae **fam. nov.** is extremely elongated (nearly twice as long as the head) and is unique among all known snakeflies (Figure 6A). Modern snakeflies, although generally entomophagous, have occasionally been observed to visit flowers and feed on pollen [3]. Interestingly, Raphidiidae, which generally have a much more elongated head and prothorax than Inocelliidae (Figure 6A), were more frequently encountered on flowers than Inocelliidae [3]. However, there has not been any study on the function of the prolonged prothorax in Raphidiidae during pollen-feeding. Nonetheless, specialized morphological traits of some Mesozoic insect pollinators have been reported to have coevolved with certain host plants [29,30]. If Juroraphidiidae **fam. nov.** possessed pollen-feeding habits, was the extreme elongated prothorax evolved for obtaining pollen from some coeval plants with deeply seated pollen chambers? This hypothesis will have to be further tested using more evidence.

## Conclusions

Juroraphidiidae **fam. nov.**, as a missing link between the suborders Priscaenigmatomorpha and Raphidiomorpha of Raphidioptera, sheds new light on the early evolution of this holometabolous order. The new family is assigned to be the sister group of the more diversified Raphidiomorpha, having typical snakefly traits. However, being a transitional lineage, it retains a number of plesiomorphic characters only shared with Priscaenigmatomorpha, which is herein considered to be firmly attributed to Raphidioptera. The diversification of snakefly suborders had occurred by the Early Jurassic. Future phylogenetic studies comprehensively combining fossil and extant taxa will further reveal the patterns of extinction and speciation in snakefly diversity.

## Methods

### Specimens examined

The specimens herein described come from the Daohugou locality. It is situated in the Ningcheng County, Neimenggu Autonomous Region, China, and belongs to the Jiulongshan Formation with a Middle Jurassic age. The fossil specimens were examined using a Leica M165C dissecting microscope and illustrated with the aid of a drawing tube. Photos of all specimens were taken by Nikon D90 and Leica DFC500 digital cameras. All fossil specimens described herein are deposited in the Beijing Museum of Natural History, Beijing (BMNH); and the Key Lab of Insect Evolution & Environmental Changes, Capital Normal University, Beijing (CNU).

### Ethical statement

No specific permits were required for us to collect the insect fossils, including the presently studied materials, from Daohugou, Ningcheng County, Neimenggu Autonomous Region, China.

### Terminology

Considering the wing venation terminology, we accept the concept based on the hypothesis of Martynov [31], which interprets M having a common stem but MA is fused with R or Rs (e.g., Aspöck et al. [27]: Figures forty-three, forty-four; Bechly & Wolf-Schwenninger [8]: Figure sixteen), and broadly follow the terminology of Aspöck et al. [27].

Wing abbreviations used in the text and figures are as follows: A, anal; C, costa; Cu, cubital; CuA, cubital anterior; CuP, cubital posterior; *ac*, anal cell; *dc*, discal cell; *doi*, discoidal cell; *m*, medial cell; M, media; MA, medial anterior; MP, medial posterior; pt, pterostigma; *r*, radial cell; R, radial; Rs, radial sector; Sc, subcosta.

### Morphological characters used in the phylogenetic analysis

1. Forewing Sc: (0) long, extending nearly to wing apex and approximating C (Figures 3 and 4); (1) short, terminating on C before pterostigma (Aspöck et al. [27]: Figure eighteen); (2) fused with R (Willmann [15]: Figures one, three).

2. Forewing with branching point of last Rs branch approximately: (0) at distal 1/3 or much more proximal (Figure 3); (1) at distal 1/4 or much more distal (Aspöck et al. [27]: Figure eighteen).

3. Forewing with Rs + MA or Rs + M branched from R approximately: (0) at proximal 1/4 or much more proximal (Figure 3; Willmann [15]: Figure three); (1) at proximal 1/3 (Willmann [15]: Figure four; Ren [32]: Figures one, six); (2) at middle (Aspöck et al. [27]: Figure eighteen).

4. Forewing MA: (0) forked near wing margin (Aspöck et al. [27]: Figure eighteen); (1) forked medially (Willmann [15]: Figure three; Ren [32]: Figures one, six); (2) simple (Figure 3).

5. Forewing with base of MA: (0) present between R and MP (Figure 3); (1) present between Rs and MP (Ren [32]: Figures one, six); (2) absent (Willmann [15]: Figures one, three).

6. Forewing Rs fused with stem of M: (0) no (Figure 3); (1) yes (Willmann [15]: Figure three).

7. Forewing Rs strongly zigzagged: (0) no (Figure 3); (1) yes (Willmann [15]: Figures one, three).

8. Forewing MP proximally fused with CuA: (0) no (Figure 3); (1) yes (Aspöck et al. [27]: Figure eighteen; Ren [32]: Figures one, six).

9. Forewing MP: (0) with two main branches, at least one of which is distally forked (Aspöck et al. [27]: Figure eighteen; Willmann [15]: Figure one); (1) with two simple main branches (Figure 3).

10. Forewing with branching point between main branches of MP: (0) at proximal 1/4 or much more proximal (Figure 3; Willmann [15]: Figures one, three); (1) at proximal 1/3 or much more distal (Aspöck et al. [27]: Figure eighteen).

11. Forewing MP zigzagged: (0) no (Figure 3); (1) yes (Aspöck et al. [27]: Figure eighteen; Willmann [13]: Figures one, three).

12. Forewing CuA and CuP: (0) having distinct common stem (Figure 3); (1) parallel and lacking common stem (Aspöck et al. [27]: Figure eighteen; Ren [32]: Figures one, six).

13. Forewing 1A: (0) forked (Willmann [15]: Figure one; Liu et al. [11]: Figure three); (1) simple (Figure 2; Aspöck et al. [27]: Figure eighteen).

14. Forewing 2A: (0) forked (Figure 3; Aspöck et al. [27]: Figure eighteen); (1) simple (Liu et al. [11]: Figure three).

15. Forewing costal region much broader than subcostal region on proximal half: (0) yes (Aspöck et al. [27]: Figure eighteen); (1) no (Figure 3).

16. Forewing with costal crossveins on distal half of costal region: (0) present (Willmann [15]: Figures one, three); (1) absent (Figure 3).

17. Forewing with 1sc-r: (0) present (Figure 3; Aspöck et al. [27]: Figure eighteen); (1) absent (Willmann [15]: Figures one, three; Liu et al. [11]: Figure three).

18. Forewing with 2sc-r: (0) present, within pterostigma (Liu et al. [11]: Figure three); (1) absent (Figure 3); (2) present, proximal to pterostigma (Aspöck et al. [27]: Figure eighteen; Ren [32]: Figures one, seven).

19. Forewing pterostigma: (0) indistinct; (1) distinct.

20. Forewing pterostigma: (0) much shorter than half of forewing length (Aspöck et al. [27]: Figure eighteen); (1) nearly as long as half of forewing (Figure 3).

21. Number of forewing radial cell: (0) 3 (Liu et al. [33]: Figure one); (1) 2 (Figure 3); (2) 4 or more (Willmann [15]: Figures one, three; Liu et al. [11]: Figure three).

22. Number of forewing discal cell: (0) 2 or more (Aspöck et al. [27]: Figure eighteen; Willmann [15]: Figures one, three); (1) 1 (Figure 3).

23. Forewing with crossveins between branches of Rs: (0) few (Figure 3); (1) many (Willmann [15]: Figures one, three; Ren [32]: Figure one).

24. Number of forewing medial cell: (0) 3 or more (Willmann [15]: Figures one, three; Ren [32]: Figure one); (1) 2 (Figure 3).

25. Forewing discoidal cells: (0) arranged into two series anteroposteriorly (Figure 3; Ren [32]: Figure six); (1) arranged into one series (Aspöck et al. [27]: Figure eighteen).

26. Number of forewing discoidal cells in anterior series: (0) 2 (Ren [32]: Figure six); (1) 3 (Aspöck et al. [27]: Figure eighteen); (2) 4 or more (Willmann [15]: Figure one; Ren [32]: Figure one); (3) 1 (Figure 3).

27. Number of forewing discoidal cells in posterior series: (0) 2 (Liu et al. [11]: Figure three); (1) 1 (Figure 3); (2) 3 or more (Willmann [15]: Figure one; Ren [32]: Figure one).

28. Forewing cua-cup: (0) present (Figure 3); (1) absent (Willmann [15]: Figure four).

29. Forewing anal cell: (0) not ovoid (Willmann [15]: Figure three; Liu et al. [11]: Figure three); (1) ovoid (Figure 2; Aspöck et al. [27]: Figure eighteen).

30. Hindwing with base of MA: (0) proximally fused with MP (Aspöck et al. [27]: Figure eighteen; Liu et al. [34]: Figure one); (1) proximally fused with R (Ren [32]: Figures one, six).

31. Hindwing with an elongate radial cell anterior to and nearly parallel with first discoidal cell: (0) no (Figure 3; Ren [32]: Figure one); (1) yes (Aspöck et al. [27]: Figure eighteen; Ren [32]: Figure, six).

32. Legs with 3^rd^ tarsomere bilobed: (0) no (Figure 1E); (1) yes (Aspöck et al. [27]: Figure sixteen).

### Phylogenetic analysis

The present analysis aimed to reveal the phylogenetic status of Juroraphidiidae **fam. nov.** in Raphidioptera. Besides Juroraphidiidae **fam. nov.**, all other valid snakefly families, i.e. Priscaenigmatidae, Baissopteridae, Mesoraphidiidae, Metaraphidiidae, Raphidiidae, and Inocelliidae, were included as ingroup taxa. Due to untested monophyly of Priscaenigmatomorpha and Priscaenigmatidae, as well as the superficial similarity between Priscaenigmatomorpha and Juroraphidiidae **fam. nov.**, all three genera of Priscaenigmatomorpha, i.e. *Chrysoraphidia* Liu, Makarkin, Yang & Ren, 2013, *Hondelagia* Bode, 1953, and *Priscaenigma* Whalley, 1985, were included and respectively coded. Scoring of the five families of Raphidiomorpha were made based on the characters of the genera *Baissoptera* Martynova, 1961, *Mesoraphidia* Martynov, 1925, *Metaraphidia* Whalley, 1985, *Turcoraphidia* Aspöck & Aspöck, 1968 and *Sininocellia* Yang, 1985 [13,24,29,30]. Nevrorthidae (Neuroptera) and Corydalidae (Megaloptera) were selected as the outgroup taxa and scoring of these two families were made based on the characters of *Sinoneurorthus* Liu, Aspöck & Aspöck, 2012 and *Jurochauliodes* Wang & Zhang, 2011 [34,35].

Totally, 32 adult morphological characters were numerically coded for 2 outgroup and 9 ingroup taxa. Morphological characters used in the phylogenetic analysis are listed in Appendix 1. 24 characters were coded as binary and 8 as multistate. Inapplicable and unavailable characters were respectively coded as “-” and “?”. The data matrix is given in Additional file 2: File S1.

The analysis was performed using WinClada ver. 1.00.08 [36] and NONA ver. 2.0 [37]. The heuristic search was used with maximum trees to keep setting to 10000 and number of replication setting to 100. An additional analysis was performed in TNT ver. 1.1 [38] with an initial New Technology search set to 100 (using a driven search with sectorial search, ratchet, drift, and tree fusing; finding the minimum tree 10 times). The branch support values were calculated with the function implemented in TNT (TBR from existing trees, retain trees suboptimal by 10 steps). All characters were treated as unordered and with equal weight. Character states were mapped on a most parsimonious tree (MPT) using WinClada ver. 1.0 [36], showing only unambiguous changes.

## Availability of supporting data

The data set supporting the results of this article is available in the TreeBASE repository, Accession URL: http://purl.org/phylo/treebase/phylows/study/TB2:S15494.

## Competing interests

The authors declare that they have no competing interests.

## Authors’ contributions

XL prepared extant and fossil material, contributed to the preparation of figures, and drafted the manuscript. DR and DY supervised palaeontological excavations, and designed the project. All authors read and approved the final manuscript.

## Supplementary Material

Additional file 1: Figure S1 Strict consensus tree of the four most parsimonious trees generated from TNT. Click here for file

Additional file 2: File S1 Data matrix for the phylogenetic analysis. Click here for file
