# Supplementary figures and images for: New transitional fossil snakeflies from China illuminate the early evolution of Raphidioptera
Source: BMC Evol Biol. 2014 Apr 18;14:84. doi: 10.1186/1471-2148-14-84 (PMC4021051; doi:10.1186/1471-2148-14-84)

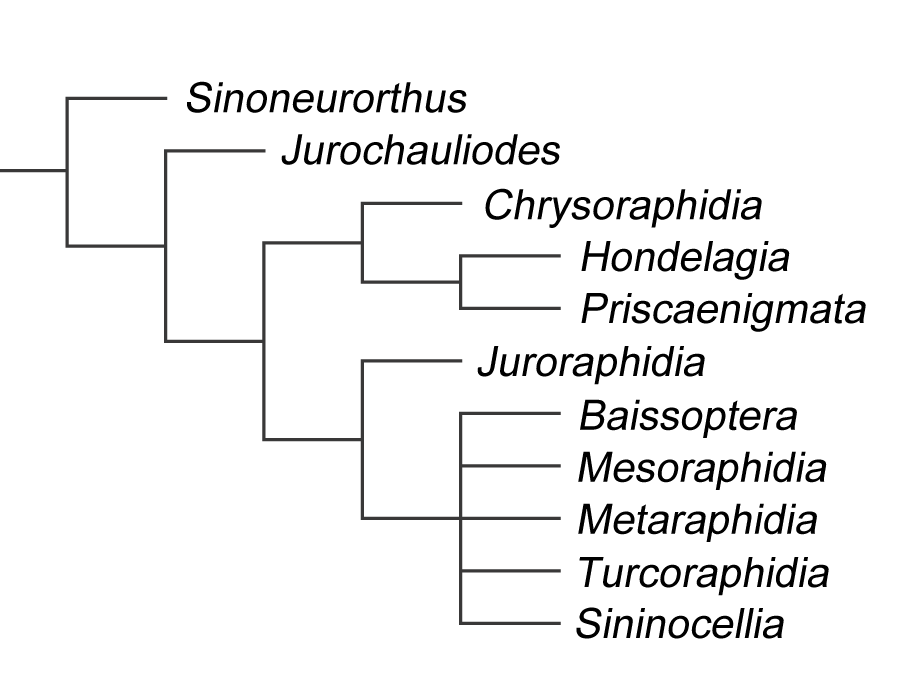

Supplement: Additional file 1: Figure S1 — Strict consensus tree of the four most parsimonious trees generated from TNT. [file 1471-2148-14-84-S1.tiff]
